# Digitalis

**Published:** 1857-03

**Authors:** 


Digitalis.—It is said that in the Dublin Hospital, this herb, in infusion, has stopped the flow in seventeen successive cases of menorrhagia. It has a great influence over the circulation, and may be useful in almost all cases of *active* hemorrhage. In what is called *passive* hemorrhage, it is less reliable than opium and acetate of lead. The dose of the infusion is an ounce to an ounce and a half.

